# The Effects of ISM1 Medium on Embryo Quality and Outcomes of IVF/ICSI Cycles

**Published:** 2013-07-31

**Authors:** Fatemeh Hassani, Poopak Eftekhari-Yazdi, Leila Karimian, Mojtaba Rezazadeh Valojerdi, Bahar Movaghar, Mohammad Fazel, Hamid Reza Fouladi, Fatemeh Shabani, Lars Johansson

**Affiliations:** 1 Department of Embryology at Reproductive Biomedicine Research Center, Royan Institute for Reproductive Biomedicine, ACECR, Tehran, Iran; 2 Department of Epidemiology and Reproductive Health at Reproductive Epidemiology Research Center, Royan Institute for Reproductive Biomedicine, ACECR, Tehran, Iran; 3 Codra Centre for Assisted Reproduction, Podgorica, Montenegro

**Keywords:** Culture Media, Embryo, Quality, Birth Weight, Babies’ lengths

## Abstract

**Background:**

The aim of this study is to investigate the effect of ISM1 culture medium on embryo development, quality and outcomes of *in vitro* fertilization/intracytoplasmic sperm injection (IVF/ICSI) cycles. This study compares culture medium commonly used in the laboratory setting for oocyte recovery and embryo development with a medium from MediCult. We have assessed the effects of these media on embryo development and newborn characteristics.

**Materials and Methods:**

In this prospective randomized study, fertilized oocytes from patients were randomly assigned to culture in ISM1 (MediCult, cycles: n=293) or routine lab culture medium (G-1TM v5; Vitrolife, cycles: n=290) according to the daily media schedule for oocyte retrieval. IVF or ICSI and embryo transfer were performed with either MediCult media or routine lab media. Embryo quality on days 2/3, cleavage, pregnancy and implantation rates, baby take home rate (BTHR), in addition to the weight and length of newborns were compared between groups.

**Results:**

There were similar cleavage rates for ISM1 (86%) vs. G-1TM v5 (88%). We observed a significantly higher percentage of excellent embryos in ISM1 (42.7%) compared to G-1TM v5 (39%, p<0.05). Babies born after culture in ISM1 had both higher birth weight (3.03 kg) and length (48.8 cm) compared to G-1TM v5 babies that had a birth weight of 2.66 kg and a length of 46.0 cm (p<0.001 for both).

**Conclusion:**

This study suggests that ISM1 is a more effective culture medium in generating higher quality embryos, which may be reflected in the characteristics of babies at birth.

## Introduction

The outcome of an assisted reproduction treatment (ART) cycle is highly dependent on numerous factors including the clinic location, equipment and disposables used (1), patient age (2, 3), stimulation regimens (4), sperm selection (5), culture condition (pH) (6), O_2_ (7), and group culture (8). It is recommended that the clinic select a suitable culture system that repeatedly produces high quality embryos for embryo transfer and cryo-preservation with low miscarriage rates that results in the delivery of a healthy baby of normal gestational age (9, 10).

*In vitro* culture and development of human embryos for days 2 or 3 transfers is performed in a variety of different culture media that range from simple to complex (11-13). Increased knowledge in the specific requirements for the development of healthy human pre-implantation embryos has resulted in the formulation of new culture media (2, 14, 15) that supports the development of embryos from fertilization to the fully expanded blastocyst stage (2, 16-24).

These products are commercially available, their quality is meticulously tested, and they are readyto- use. Therefore the use of these high quality products should significantly improve embryo quality, cost-efficiency of the treatment, and the clinic’s reputation (12).

The purpose of this study was to compare the efficiency of two different commercially available media products (ISM1, MediCult, Denmark vs. G-1TM v5, Vitrolife, Sweden) in supporting the development of embryos (days 2 and 3), their ability to implant and deliver a healthy baby at term.

## Materials and Methods

### Patient population

This prospective randomized study was performed during a five month period (February- June, 2009). During this period all culture conditions, routines and disposables were kept constant in the laboratory. On the day of oocyte pick up, there were 583 patients randomized to both groups according to a randomization list based on sequential numbers in sealed envelopes. The ISM1 group (experimental) consisted of 293 patients compared to 290 patients in the routine lab procedure group (control). Depending on the causes of infertility, patients received either standard *in vitro* fertilization (IVF) or intracytoplasmic sperm injection (ICSI). Excluded from the study were patients with previous fertilization failure [19 (experimental) vs. 8 (control)]; those with no oocytes retrieved [1 (experimental) vs. 1 (control)]; and patients diagnosed with ovarian hyperstimulation hormone syndrome [OHSS; 22 (experimental) vs. 18 (control)].

Inclusion criteria were counseling patients undergoing ICSI treatment, between 18 to 40 years of age who had a minimum of 2 follicles (≥18 mm) at the time of oocyte collection.

Patients were randomly assigned to have their fertilized oocytes cultured in ISM1 (MediCult, cycles: n=293, written informed consent was taken from all the couples who received this culture medium) or routine lab procedure (G-1TM v5; vitrolife, cycles: n=290) according to the daily medium schedule for oocyte retrieval. This study was approved by Ethical Committee of Royan Institute.

### Stimulation regimes and oocyte retrieval techniques

Down regulation and ovarian stimulation were performed in accordance with a previously reported stimulation regimen (25). In brief, pituitary gonadotropin secretion was suppressed with the GnRH agonist buserelin acetate (Suprefact, Hoechst AG, Germany) via subcutaneous (SC) injection (500 mg/d) or by nasal spray (800 mg/d) starting in the mid luteal phase of the preceding ovarian cycle (day 21).

Once ovarian suppression was confirmed (serum E2 ≤50 pg, FSH ≤12 IU and LH ≤5 IU), ovarian stimulation was initiated with recombinant FSH (Gonal F; SC injection, 150 IU/day, Serono, Switzerland). The dose was adjusted according to the response of the ovarian follicular development and monitored by serial vaginal ultrasonography. When at least three follicles reached 18 mm in diameter, both GnRH agonist and hMG were discontinued and a single dose of hCG (10000 IU; Pregnyl; Organon, Netherlands) was administered.

Oocyte retrieval was performed via vaginal ultrasound- guided follicle aspiration, 36-38 hours after administration of hCG.

### Intracytoplasmic sperm injection (ICSI)

After egg collection, the cumulus-oocyte complexes were either placed in 50 μL droplets of Universal IVF medium and covered with liquid paraffin (MediCult, Denmark) as the experimental group or in 50 μL droplets of Ham’s F-10, (Biochrom AG; Berlin, Germany), which had been supplemented with 10% recombinant human serum albumin (rHA, Vitrolife) and covered with pre-incubated mineral oil (Sigma, St. Louis, MO), as the control group. Oocyte denudation was performed with ICSI Cumulase (MediCult) in the experimental group or with 80 IU of hyaluronidase, (Sigma, USA) in the control group, two hours after egg collection. Sperms were immobilized in 10% PVP droplets (MediCult) in the experimental group and in10% PVP droplets (Global) in the control group by breaking the tail by an injection pipette. Then, a single sperm with head front was injected into each oocyte that had a visible first polar body (MII) (26, 27). All semen samples were prepared by swim-up method.

### Control of fertilization and embryo culture

The fertilization rate was controlled 18-20 hours after sperm injection. Normally fertilized oocytes that had evidence of two pronuclei were washed and transferred to overnight pre-incubated in 20 μl droplets of ISM1 or G- 1TM v5 supplemented with 10% recombinant human serum albumin in the experimental and control groups respectivly and covered with either pre-incubated liquid paraffin (experimental group) or mineral oil (control group). All cultures incubated at 37˚C, in 5% O_2_ and 6% CO_2_.

### Embryo scoring and transfer techniques

On the day of embryo transfer (44-72 hours after injection), we scored embryo morphology according to the following quality criteria:

**Excellent**
Day 2: 2-4 even size blastomeres with ≤10% fragmentation
Day 3: 6-8 even size blastomeres with ≤10% fragmentation
**Good**
Day 2: 2-4 even or uneven size blastomeres with 10%-20% fragmentation
Day 3: 6-8 even or uneven size blastomeres with 10%-20% fragmentation
**Poor**
Uneven and few blastomeres with >20% fragmentation

Based upon the embryo quality, female age and number of previous cycles, we selected a maximum of four high quality embryos and incubated them in UTM (MediCult) for the experimental group or Embryo Glue (Vitrolife) for the control group, for a period of 20-120 minutes before embryo transfer.

All embryo transfers were performed with a Labotect catheter (Labotect, Germany) against a filled urine bladder with the expertise of gynecologists and embryologists.

### Statistical analysis

We used the Statistical Package for the Social Sciences (SPSS 11.5; Chicago, IL, USA, http:// www.spss.com) software to analyze differences amongst the variables of both groups by Chisquare and Fisher’s exact tests for categorical variables or the student’s t test for continuous variables, as appropriate. A p value <0.05 was considered to be statistically significant.

## Results

Patient characteristics of the two groups are summarized in table 1. The number of cycles, mean female age, indications for infertility, number of previous treatment cycles, retrieved oocytes and percentage of cleaved embryos were similar in both groups.

There were no significant differences between the groups in terms of cleavage rates, number of embryos transferred, clinical pregnancy and implantation rates and multiple pregnancies. There were a significantly higher percentage of excellent embryos in the experimental group (42.7%; ISM1) compared to the control group (39%, p<0.05). There was also a trend towards higher clinical and multiple pregnancy rates as well as implantation rates (Table 2) for the experimental group.

In terms of abortion, the experimental group (20.5%) was not significantly different from the control group (21.1%). There was no significant difference in baby take home rates (BTHR) between both groups, however both the length [48.8 cm (experimental) vs. 46.0 cm (control)] and weight [3.03 kg (experimental) vs. 2.66 kg (control)] of babies born were significantly higher in the expreimental group (p<0.001 for both parameters).

**Table 1:** Patients’ ART cycle characteristics in the MediCult (experimental) and lab routine media (control)


Parameters	Experimental group	Control group	P value

**No. of cycles**	293	290	0.37
**Female age (Y) (mean ± SD)**	32.4 ± 5.7	31.8 ± 5.6	0.18
**Male factor infertility**	170/293 (58%)	170/290 (58.6%)	0.88
**Female factor infertility**	66/293 (22.5%)	62/290 (21.4%)	0.73
** Mixed infertility**	44/293 (15%)	46/290 (16%)	0.77
**Unexplained infertility**	13/293 (4.4%)	12/290 (4.1%)	0.83
**Previous ART cycles (mean ± SD)**	1.7 ± 1.2	1.8 ± 1.0	0.21
** No. of retrieved oocytes (mean ± SD)**	9.1 ± 5.6	8.8 ± 5.1	0.52


**Table 2:** Fertilization, cleaved embryo, embryo quality and clinical pregnancy in the MediCult (experimental) and lab routine media (control) groups


Parameters	Experimental group	Control group	*P value

**Cleavage rate**	86% (1471/1706)	88% (1455/1678)	0.68
**Embryo quality**			
**Excellent**	43% (628)	39% (567)	0.04a
**Good**	39% (580)	41% (599)	0.33
**Poor**	18% (261)	20% (288)	0.15
**No. of transferred embryos (mean ± SD)**	2.4 ± 0.8	2.4 ± 0.8	0.83
**Clinical pregnancy rate/embryo transfer**	32.1% (94/293)	27.6% (80/290)	0.23
**Implantation rate**	15% (102/697)	12% (84/693)	0.16
**Multiple pregnancy rate**	8.5% (8/94)	3.8% (3/80)	0.19
**Single**	91.5% (86/94)	96.2% (77/80)	0.19
**Twin**	8.5% (8/94)	2.5% (2/80)	0.09
**Triplet**	0% (0/94)	1.3% (1/80)	0.46


a; Statistically significant differences between the two groups and *; Fisher’s exact test.

**Table 3:** Clinical outcome, abortion and take-home baby rate in the MediCult (experimental) and lab routine media (control) groups


Parameters	Experimental group	Control group	*P value

**Abortion rate**	20.5% (18/88)	21.1% (16/76)	0.9
**First trimester **	77.8% (14/18)	87.5% (14/16)	0.58
**Second trimester **	16.7% (3/18)	12.5% (2/16)	0.48
**Third trimester **	5.5% (1/18)	0% (0/16)	0.35
**Baby take home rate (BTHR) **	84.3% (86/102)	80.4% (78/97)	0.57
**Multiple pregnancy rate**	8.5% (8/94)	3.8% (3/80)	0.19
**Single **	78.9% (56/71)	66.7% (40/60)	0.1
**Twin **	21.1% (15/71)	31.7% (19/60)	0.1
**Triplet**	0% (0/71)	1.7% (1/60)	0.1
**Sex**			
**Male**	44.1% (38/86)	51.3% (40/78)	0.3
**Female**	55.8% (48/86)	48.7% (38/78)	0.3
**Length (cm, mean ± SD)**	48.8 ± 0.52	46.0 ± 0.66	0.001 a
**Weight (kg, mean ± D)**	3.03 ± 0.07	2.66 ± 0.08	0.001 a


a; Statistically significant differences between the two groups and *; t test. Of all pregnant patients, 10 (n=6 for expreimental group and n=4 for control group) patients were not available and were excluded from table 3.

## Discussion

There are a variety of different *in vitro* culture systems available for laboratory procedures applied to gamete maintenance and embryonic growth. These media products range from simple solutions to more complex culture media systems, which have been adapted to the changes that the developing embryos face during their passage through the female reproductive tract (11-13). Many recent discoveries have been fundamental in describing different biochemical, physiologic, genetic, and epigenetic characteristics of the embryo, resulting in recent innovations in this field. Culture media formulations are presumed to be of essential importance for normal development of human embryos in the controlled environment of an incubatorin addition to their effects on developing embryos (22, 23, 28).

A general agreement does not exist regarding the efficacy of different commercially available *in vitro* culture media, since some studies have reported no differences in embryo quality, pregnancy and implantation rates (18, 21, 29), whereas others have observed subtle differences in day 3 embryo quality [G1.2 vs. Sydney IVF sequential media (16), G1.2 vs. HTF (20) and GIII series versus ISM1(30)].

Several studies have investigated the effect of ISM1 and G-1TM v5 media on embryo quality and implantation rate. Embryo morphology on days 2 and 3 significantly enhanced when the embryos were cultured in GIII series versus ISM1 (18). Another study showed that "in a day 3 embryo transfer program, G1.2/G2.2 media were superior to Sydney IVF media, whereas both media yielded similar outcomes in blastocyst transfer program" (20).

A prospective study on sibling oocytes showed that embryo quality in the day 2 stage improved under ISM1 culture rather than FertiCult culture medium, even if data regarding pregnancy and implantation rate were not reported (31). Another study demonstrated that ISM1 culture medium seemed to improve the performance of embryonic growth and development, as well as increased the percentage of pregnancies (32).

For this reason, we conducted a prospective randomized trial at Royan Institute to compare embryo development parameters, IVF outcome and BTHR at the end of the gestational period between ISM1 and G-1TM v5 media, which is used routinely in our laboratory.

The result of the study suggests that both ISM1 and G-1TM v5 support embryo development *in vitro*, whereas culture in ISM1 gives a higher proportion of excellent quality embryos.

Good embryo quality, cleavage rate, and single, triplet pregnancy rate in the control group were higher than ISM1 medium, but there were no significant differences. In ISM1, the implantation, clinical pregnancy and twin pregnancy rates were higher than the G-1TM v5 medium. However, this difference was not significant. The percentage of excellent embryos was significantly higher than the control group which might be reflected in the characteristics of babies born. Babies born after culture in ISM1 had both significantly higher birth weight and were also longer compared to the control group, both of which were significant.

In vivo as well as *in vitro* fundamental factors for proper embryonic development could be summarized as oxygen consumption, the balance between reactive oxygen species and antioxidant defense, energetic metabolites (Na^+^, K^+^, adenosine triphosphatase), and amino acid turnover. The oxygen requirement appears constant over the course of the early embryonic development phases up to the blastocyst stage, and later increases (33).

Although in vivo physiologic reactive oxygen species production is controlled continuously by a strong cellular defense, the lack of this system during the *in vitro* culture could cause oxidation of proteins, lipids, DNA, and the misregulation of metabolic pathways that may lead to cell death (34-36). On the basis of these observations, it can be speculated that the antioxidant elements present in ISM1 culture medium sustain the cultured embryos, thereby avoiding developmental failure and also achieving the best quality embryos for implantation (32). The result of this study has demonstrated that ISM1 medium improved embryo development. The percentage of excellent embryos was significantly higher in ISM1 medium compared with the control group (G-1TM v5). This result was possibly due to the antioxidant elements present in ISM1 culture medium.

Singleton children born after IVF have a significantly lower birth weight compared with their spontaneously conceived peers (37-39), even after adjustments for maternal age and parity. This phenomenon has produced concern, because low birth weight is associated with morbidity over both the short and long term (40). Speculations about possible causes, including epigenetic alterations by ovarian stimulation, have been published (41). Theoretically the lower birth weight in IVF singletons could be attributed to i. maternal and/or paternal characteristics associated with infertility; ii. the effects of ovarian stimulation on oocytes, endometrium or the endocrinology of the luteal phase or early pregnancy; or iii. IVF laboratory procedures such as ICSI or embryo culture conditions (42, 43). The implementation of suitable quality control has focused on the effectiveness of culture media in improving embryo morphology and it is fundamental to the success of an IVF laboratory (44).

"The possible interference of assisted reproduction techniques (ART) with epigenetic reprogramming during early embryo development has recently sparked renewed interest about the reported lower birth weight among infants born as a consequence of infertility treatments" (45).

Both the shorter duration of pregnancy and the reduced neonatal birth weight could be caused by inactivation of paternal alleles during the imprinting process. If the shorter duration of pregnancy and the lower birth weight after IVF and ICSI can be attributed to a distinct defect of epigenetic phenomena at work during parental imprinting, then this defect may have later effects on the individual (46, 47) and has the potential to be transferred to other generations (48).

ISM1 culture medium was formulated to promote correct imprinting for ensuring correct development and implantation (32). As demonstrated by Cassuto et al. (49), different timing and culture conditions can have different effects on embryo development and implantation rate. Extended culture time and particular culture conditions result in an increase in monozygotic twinning. In this study the use of only ISM1/ISM2 culture conditions resulted in lower monozygotic twin pregnancies, independent of the technology used (IVF/ICSI) (49). The result of our study demonstrated that babies born after culture in ISM1 had both higher birth weight and were also longer than those in control group. This result might be due to correct imprinting that ensured correct development and implantation.

## Conclusion

This study suggests that on the one hand ISM1 contains antioxidant elements and on the other hand it is formulated to promote correct imprinting to ensure correct development and implantation. ISM1 is presumed to be a more effective culture medium that generates higher quality embryos, which is reflected in the characteristics of babies born. Since numerous factors that include mother’s diabetes and multiple pregnancies, among others may also impact the height and the weight of the newborn, we cannot directly relate the change in these two characteristics solely to the culture media. For reaching a definitive conclusion, additional studies that consider these factors are warranted.
